# Biological invasion of oxeye daisy (*Leucanthemum vulgare*) in North America: Pre-adaptation, post-introduction evolution, or both?

**DOI:** 10.1371/journal.pone.0190705

**Published:** 2018-01-04

**Authors:** Sonja Stutz, Patrik Mráz, Hariet L. Hinz, Heinz Müller-Schärer, Urs Schaffner

**Affiliations:** 1 CABI, Delémont, Switzerland; 2 Department of Biology/Ecology & Evolution, University of Fribourg, Fribourg, Switzerland; 3 Herbarium and Department of Botany, Charles University in Prague, Prague, Czech Republic; Universidade da Coruna, SPAIN

## Abstract

Species may become invasive after introduction to a new range because phenotypic traits pre-adapt them to spread and become dominant. In addition, adaptation to novel selection pressures in the introduced range may further increase their potential to become invasive. The diploid *Leucanthemum vulgare* and the tetraploid *L*. *ircutianum* are native to Eurasia and have been introduced to North America, but only *L*. *vulgare* has become invasive. To investigate whether phenotypic differences between the two species in Eurasia could explain the higher abundance of *L*. *vulgare* in North America and whether rapid evolution in the introduced range may have contributed to its invasion success, we grew 20 *L*. *vulgare* and 21 *L*. *ircutianum* populations from Eurasia and 21 *L*. *vulgare* populations from North America under standardized conditions and recorded performance and functional traits. In addition, we recorded morphological traits to investigate whether the two closely related species can be clearly distinguished by morphological means and to what extent morphological traits have changed in *L*. *vulgare* post-introduction. We found pronounced phenotypic differences between *L*. *vulgare* and *L*. *ircutianum* from the native range as well as between *L*. *vulgare* from the native and introduced ranges. The two species differed significantly in morphology but only moderately in functional or performance traits that could have explained the higher invasion success of *L*. *vulgare* in North America. In contrast, leaf morphology was similar between *L*. *vulgare* from the native and introduced range, but plants from North America flowered later, were larger and had more and larger flower heads than those from Eurasia. In summary, we found litte evidence that specific traits of *L*. *vulgare* may have pre-adapted this species to become more invasive than *L*. *ircutianum*, but our results indicate that rapid evolution in the introduced range likely contributed to the invasion success of *L*. *vulgare*.

## Introduction

Why only certain species become invasive when introduced into new ranges and others not is still a major question in invasion biology. Several studies focused on identifying traits that may pre-adapt plant species to become invasive after introduction to a new range (e.g. [1–3]). Studies comparing invasive with non-invasive introduced plant species revealed that traits associated with high plant performance such as a larger size or biomass [3–5], faster growth [6–8], or a higher specific leaf area (SLA) [5–7, 9, 10] may pre-adapt species to become invasive. Beside pre-adaptation, evolutionary changes in response to novel selection pressures in the introduced range may also play an important role in enabling introduced plants to become invasive [11–16]. The most common hypothesis regarding rapid evolution in the introduced range is the Evolution of Increased Competitive Ability (EICA) hypothesis [17], which proposes that certain plants may adapt to a reduced herbivore pressure in the introduced range by re-allocating resources from defense to growth and reproduction. However, introduced plants may also adapt to other biotic (e.g. plant competition) or abiotic (e.g. climate [11, 13, 18]) factors that differ from their native range. Numerous studies have investigated whether there are evolutionary changes in traits related to performance by growing plants from the native and introduced range under standardized conditions and these studies generally found evolution of increased size but only moderate or no support for the EICA hypothesis (reviewed by [14, 19]). In contrast, only a few studies have examined whether there is also rapid evolution in other plant traits [13, 18, 20–24]. For example, a recent study comparing herbarium specimens of species invasive to Australia showed that five out of 19 species have undergone significant changes in leaf shape through time [13].

The diploid *Leucanthemum vulgare* (Vaill.) Lam. (2*n* = 2*x* = 18) and the closely related tetraploid *Leucanthemum ircutianum* DC. (2*n* = 4*x* = 36) are perennial herbs native to Europe and western Asia. In the native range, both species are mainly found in meadows and pastures and can also be found in ruderal habitats such as roadside areas, but they usually do not persist on sites not mown for several years. Both species have been introduced as ornamentals and seed contaminants to North America and have become naturalized, but only the diploid *L*. *vulgare* has become invasive [25–27]. In its introduced range, *L*. *vulgare* invades pastures, meadows, roadside areas and forest openings where it reduces plant species diversity and hay or forage production [28, 29]. In North America, it was reported to be naturalized in Québec and in the north-eastern USA by the 18th century [25, 30]. Nowadays, it is common in the north-eastern and north-western states of the USA and in the south-eastern and south-western provinces of Canada [28, 29]. In a recent ploidy screening of 98 *Leucanthemum* populations collected across North America only two populations have been identified as *L*. *ircutianum*, all others were *L*. *vulgare* [27]. The higher invasion success of *L*. *vulgare* compared to *L*. *ircutianum* is unlikely caused by differences in the introduction rates as both species largely overlap in their native range distribution where *L*. *ircutianum* is more common than *L*. *vulgare* [31–33]. In addition, the analyses of 13 seed sources commercially sold under the name *L*. *vulgare* by twelve US and one Canadian seed company revealed that twelve contained *L*. *ircutianum* seeds and only one *L*. *vulgare* seeds (S1 Appendix). Moreover, the higher invasion success of the diploid *L*. *vulgare* is in contrast to recent studies that found a positive association between polyploidy and invasiveness in plants [34–36].

Due to their similar morphology, *L*. *vulgare* and *L*. *ircutianum* have often been treated as a species complex (sometimes referred to as “*L*. *vulgare* s.l.”), together with other morphologically similar species [37–39]. *Leucanthemum ircutianum* is an allopolyploid species with *L*. *vulgare* and possibly *L*. *virgatum* (Desr.) Clos as parental species [33]. Several attempts have been made to morphologically characterize *L*. *vulgare* and *L*. *ircutianum*in in their native range (e.g. [31, 40–43]). Differences in leaf shape such as the size of the teeth or lobes at the base of the stem leaves or the length to width ratio of the stem leaves have been identified that help to distinguish the two species from each other [31, 40–43], but phenotypic variation between and even within populations was also found to be high [42, 43].

In this study, we compared performance, functional and morphological traits of 20 native (Eurasian) *L*. *vulgare* populations with 21 native *L*. *ircutianum* populations and with 21 introduced (North American) *L*. *vulgare* populations under standardized conditions. We defined performance traits as traits that directly contribute to fitness (e.g. biomass, number of flower heads), functional traits as traits that impact fitness indirectly (e.g. specific leaf area, time to flowering [44]) and morphological traits as traits potentially important for species determination (e.g. leaf shape). With the comparison of native *L*. *vulgare* and *L*. *ircutianum* we aimed to investigate whether the two species differ in performance and functional traits that may explain the higher invasion success of *L*. *vulgare* in North America and whether they can be clearly distinguished by morphology. With the comparison of *L*. *vulgare* from the native and introduced range we aimed to determine whether *L*. *vulgare* from the two ranges differ in phenotypic traits, which would suggest that rapid evolutionary changes after the introduction to North America may have further increased the invasive potential of *L*. *vulgare*. We hypothesized that *L*. *vulgare* possesses performance and functional traits that made it better pre-adapted to become invasive in North America than *L*. *ircutianum*, and that some of these performance and functional traits have evolved post-introduction to further increase the invasiveness of *L*. *vulgare*. Within the restriction that little is known about evolutionary changes in morphological traits of invasive plants, we predicted that morphological traits have less significantly changed post-introduction than performance and functional traits, because the latter are likely to be under stronger selection.

## Materials and methods

### Cultivation of plant material

**Fig 1:**
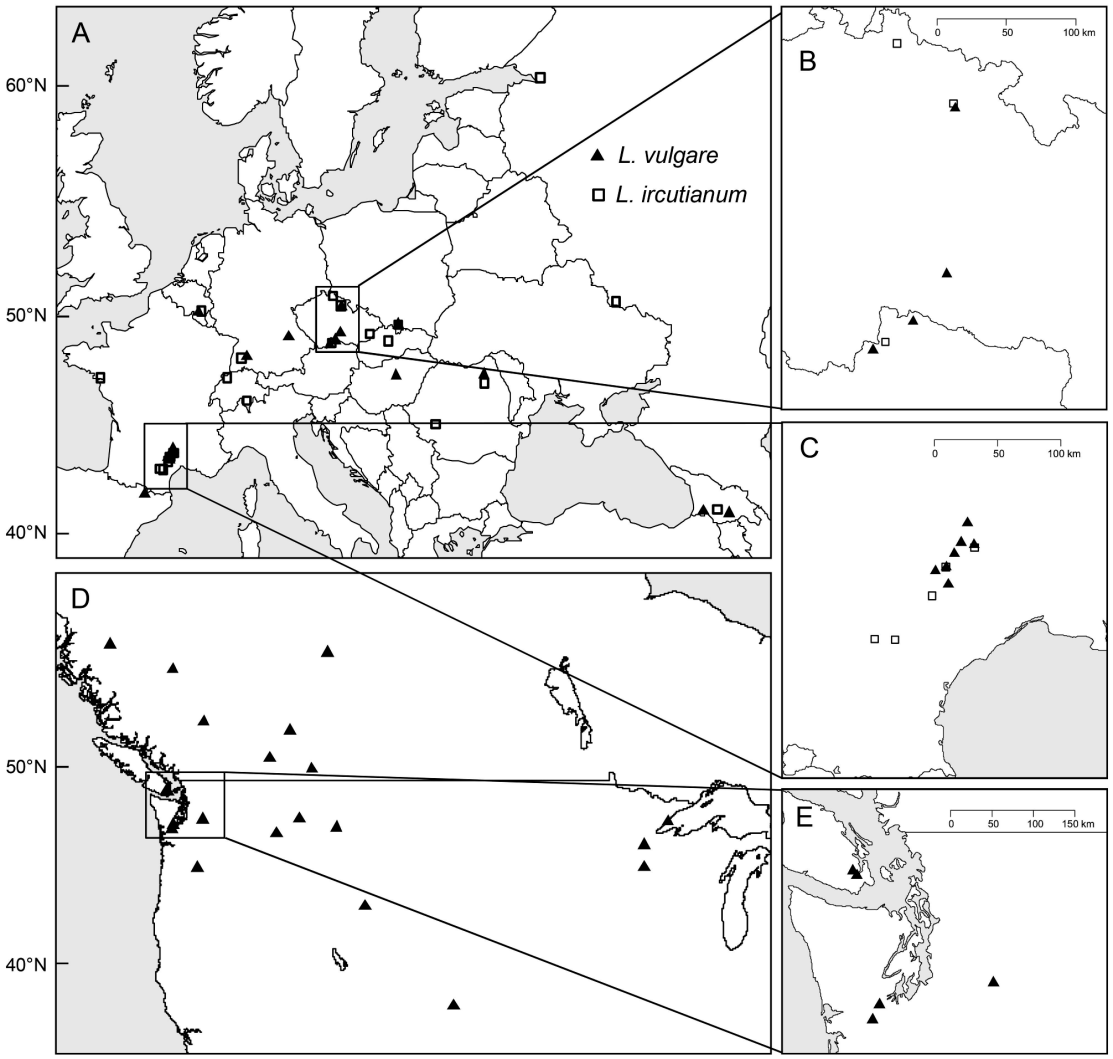
Sampling locations of *Leucanthemum vulgare* (triangles) and *L*. *ircutianum* (squares) populations from the (A, B, C) native (Eurasia) and (D, E) introduced (North America) range.

The seeds used in this experiment were collected from field populations from 2009 to 2013 and stored in paper bags at 2°C until sowing. To distinguish between the morphologically similar *L*. *vulgare* and *L*. *ircutianum* the ploidy level of each population used in the experiment was determined prior to the experiment either as part of a previous study [27] or as part of this study. For this purpose flow cytometric analyses (CyFlow SL Green 2P, Partec) were performed on one or two bulk samples per population each containing two seeds from up to five maternal plants per population (see [27] for more information on the methods). In one European population (PL3) diploid and tetraploid plants were detected and seeds from individual plants were analysed separately. *Leucanthemum vulgare* and *L*. *ircutianum* plants from this population were subsequently treated separately (PL3vul and PL3irc). In total, we grew potted plants of 20 *L*. *vulgare* and 21 *L*. *ircutianum* populations from the native range (Europe and western Asia) and 21 *L*. *vulgare* from the introduced range (USA and Canada) under standardized conditions in a common garden at CABI in Delémont, Switzerland (47.3732°N, 7.3261°E; 515 m) (Fig 1, S2 Appendix). Each population was represented by ten plants grown from seeds of five maternal plants.

From 4 to 8 November 2013, six seeds from five maternal plants per population were sown in 4 cm x 4 cm cells of seedling trays. Three seeds were sown per cell, resulting in a total of 620 cells. The seedling trays were filled with a mixture of garden soil (Selmaterra, Eric Schweizer AG, Switzerland), sand and vermiculite (14:3:1) with 1 g/L of slow-release NPK fertiliser (Hauert Tardit 6M) added. After germination, the numbers of seedlings were reduced to one per cell, i.e. two per maternal plant. To ensure that each population was represented by ten seedlings, missing seedlings (n = 13) were replaced by seedlings of the corresponding population. The seedlings were kept in a heated greenhouse with artificial light (16h photoperiod, temperature constantly set at 20°C for four weeks and then decreased to 15°C during the night) until the beginning of January 2014 and then moved to a colder greenhouse (no artificial light, temperatures maintained above 0°C) for vernalization in order to promote flowering in the first year. At the beginning of February, all 620 plants were potted in 1 L pots filled with the same mixture of soil as described above and kept in the same greenhouse for another two weeks. Afterwards, all plants were moved outside in a garden bed (6 m x 6 m), where they were embedded in sawdust and arranged in a random design (distance between pots: 6 cm). The plants were exposed to precipitation and watered during dry periods. All plants survived throughout the experiment and all except six plants flowered during the summer.

A collection permit was obtained to collect *L*. *vulgare* seeds in the Banff National Park of Canada (Permit Number: BAN-2011-9993). No specific permissions were needed for seed collections at other sites since they were not in protected areas. No permits were necessary to introduce the seeds to Switzerland. The study was carried out on private land with the permission of the owner.

### Phenotypic measurements

From November 2013 to July 2014, a total of 17 phenotypic traits were measured, recorded or calculated (Table 1; for reasons for inclusion see S3 Appendix). Beside seven traits directly related to vegetative and reproductive performance (germination rate, number of leaves and the length of the blade of the longest leaf at the seedling stage as well as the total biomass, the length of the longest shoot and the number of shoots and flower heads at the end of the flowering stage) and four functional traits (days to germination, days to flowering, specific leaf area (SLA) and leaf dry matter content (LDMC)) we also recorded six morphological traits potentially important to distinguish *L*. *vulgare* and *L*. *ircutianum* as suggested by previous authors [32, 40, 43] and our own field observations (the length to width ratio of the leaf blade at the seedling stage and of a mid-stem leaf at the flowering stage, the perimeter to area ratio of rosette and mid-stem leaves, the width of the undivided middle part at the base devided by the total width at the base of a randomly chosen stem leaf and flower head diameter). Although we classified flower head diameter as a morphological trait we acknowledge that it may potentially also be correlated with pollination and seed production and could therefore also be classified as a performance trait.

**Table 1:** List of phenotypic traits recorded for *Leucanthemum vulgare* and *L*. *ircutianum*.

**Performance traits**
Germination rate
Length of leaf blade of longest leaf of three-month old seedlings
Number of leaves longer than 1 cm of three-month old seedlings
Number of shoots at the end of the flowering stage
Number of flower heads at the end of the flowering stage
Length of longest shoot at the end of the flowering stage
Dry weight of above ground biomass at the end of the flowering stage
**Functional traits**
Days to germination
Days to flowering
Leaf dry matter content: ratio of dry to fresh weight of a randomly chosen rosette leaf
Specific leaf area: ratio of area to dry weight of a randomly chosen rosette leaf
**Morphological traits**
Length to width ratio of leaf blade of longest leaf of three-month old seedlings
Perimeter to area ratio of randomly chosen rosette leaf
Perimeter to area ratio of randomly chosen mid-stem leaf
Length to width ratio of leaf blade of longest leaf of three-month old seedlings
Width of undivided middle part at the base devided by the total width at the base of a randomly chosen stem leaf
Flower head diameter (average taken from three flower heads per plant)

From 11 November to 4 December, germination was monitored two to three times a week and the date when the first seedling emerged was recorded for each cell of the seedling trays. The germination rate was then calculated per population as the number of seeds that germinated, divided by the number of seeds that had been sown. In early February, just before transplanting the seedlings into the 1 L pots, the number of leaves longer than 1 cm were recorded and the length of the longest leaf as well as the length and width of its leaf blade were measured using a calliper with 1 mm-precision. At the end of April, when all plants were still in the rosette stage, one fully expanded and undamaged leaf was collected from each plant and immediately weighed. For subsequent measurements a binary image was taken from each leaf using a flatbed scanner. Afterwards, all scanned leaves were dried at 80°C for 24h and weighed. The dry to fresh weight ratio of the rosette leaves was used to calculate LDMC and the leaf area to dry weight ratio was used to calculate SLA. From April to July the onset of flowering, defined as the date when the first flower head of a plant was completely open, was recorded two to three times per week. Once at least three principal flower heads of a plant were completely open (typically 1–2 weeks after the first flower head opened) the diameter of three principal flower heads was measured using a calliper with 0.1 mm-precision. At the same time, an undamaged mid-stem leaf of a fully developed stem was removed and scanned with a flatbed scanner for subsequent morphological measurements. As soon as more than half of the flower heads of an individual plant started to senesce, which corresponded with the date when all or the majority of the flower heads were completely open and no new shoots were produced, the total number of stems and flower heads as well as the length of the longest stem were recorded. At the same time, all above ground biomass was harvested, dried for at least 48h at 80°C and then weighed.

Several morphological measurements were taken on the scanned rosette and stem leaves using the software ImageJ [45]. For each of the scanned rosette and stem leaves the area and perimeter were measured and their ratio was calculated. In addition, the total leaf length, the leaf width (not including teeth and lobes) at the leaf length midpoint, the total leaf width (including teeth and lobes) at the leaf base and the width of the undivided middle part (excluding lobes and teeth) at the leaf base were measured on the stem leaves as pictured in S4 Appendix. These measurements were then used to calculate the leaf length to width ratio and the ratio of the undivided middle part to the total width at the leaf base (a measurement to describe the relative length of the teeth and lobes at the base of the stem leaf).

### Statistical analyses

In a first step, two principal component analyses (PCAs) based on the correlation matrices of individual plants and population means of all recorded traits (Table 1) were performed to visualize the relationships among native and introduced *L*. *vulgare* as well as native *L*. *ircutianum* plants and populations. Plants with missing values for one of the measured traits were excluded from the PCA based on individual plants. This reduced the number of plants included in this PCA to 556 (192 *L*. *ircutianum* plants, 168 *L*. *vulgare* plants from Eurasia and 196 *L*. *vulgare* plants from North America).

In a second step, linear mixed models (for continuous variables) and generalized linear mixed models (for discrete variables) were used to test for differences in individual plant traits between *L*. *vulgare* and *L*. *ircutianum* from the native range and between *L*. *vulgare* from the native and introduced range. For each plant trait, separate analyses were conducted to compare native *L*. *vulgare* with native *L*. *ircutianum* and native with introduced *L*. *vulgare* populations. Continuous variables were transformed as required to address normality assumptions. Maternal plant nested within population was considered as a random factor. To analyse differences in germination rates a quasi-binomial generalized linear model was used. To assess whether differences in storage time affected germination rate the year when the seeds had been collected was included in the model. Since storage time did not have an influence on the germination rate (*t* = 0.9, *P* = 0.4), it was subsequently removed from the analyses. To analyse whether the biomass at the end of the flowering stage was correlated with the days to flowering and whether there was a correlation between latitude of population origin and days to flowering or biomass we conducted linear regression analyses on population means. These analyses were done separately for native and introduced *L*. *vulgare* populations.

All analyses were performed with the software R version 3.2.3 [46]. The PCAs were done using the function dudi.pca in the ade4 package [47], linear mixed models were done using the function lme in the nlme package [48] and generalized linear mixed models were done using the function glmer in the lme4 package [49].

## Results

**Fig 2:**
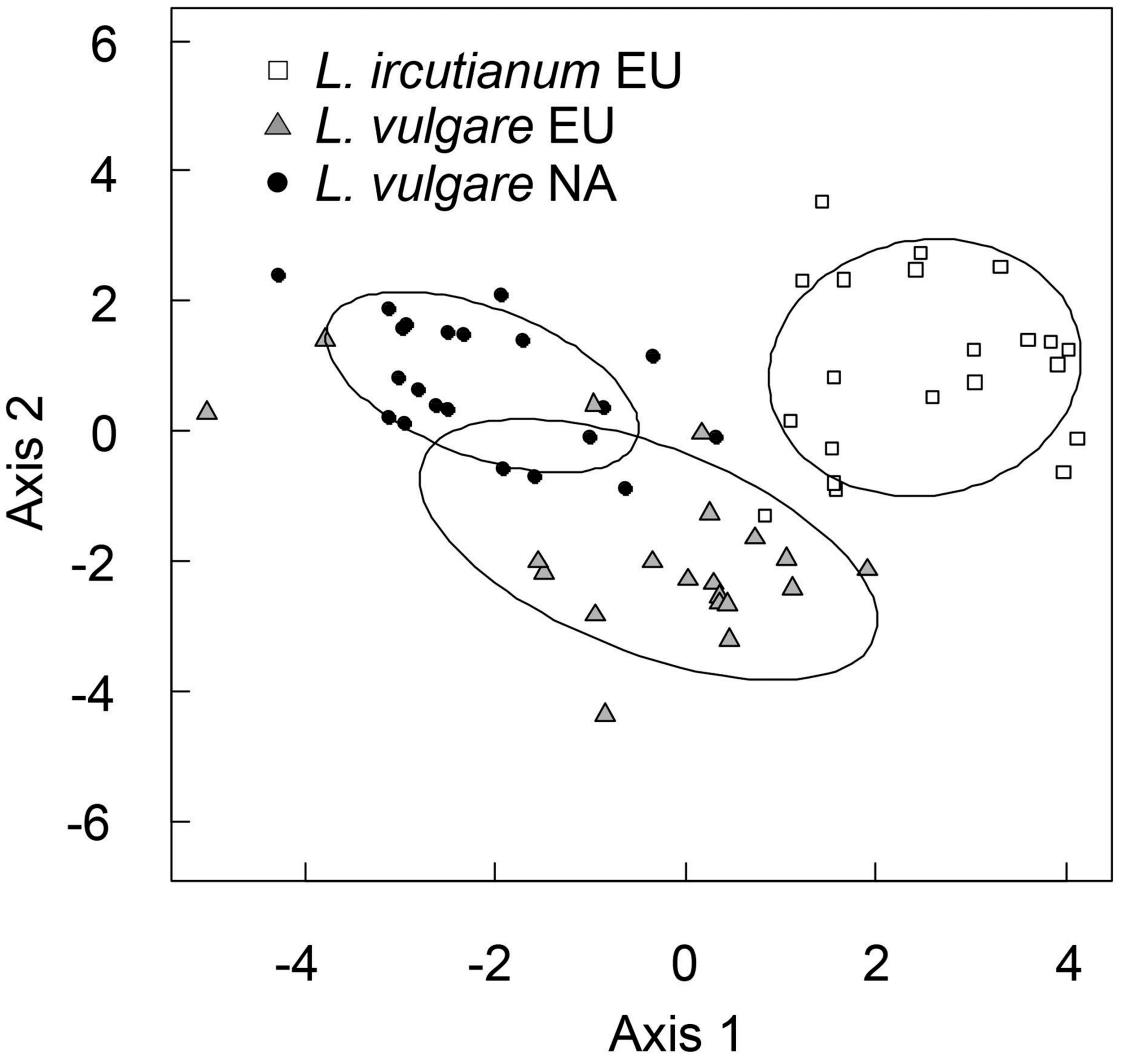
Principal Component Analysis (PCA) plot based on population mean values of 17 traits measured from ten plants from 62 populations of *Leucanthemum vulgare* and *L*. *ircutianum* from the native (Eurasia, EU) and introduced (North America, NA) range grown under standardized conditions. To show the phenotypic space occupied by each species from the native and introduced range confidence ellipses defined by the gravity center (centroid) of the cloud and 1.5 times the standard deviation were constructed. The first axis explains 31.7% of the total variation in the dataset; the second axis explains 17.9%.

The PCA based on individual plants revealed a relatively clear separation of *L*. *vulgare* and *L*. *ircutianum* although the variability was large and some *L*. *vulgare* plants grouped with *L*. *ircutianum* and vice versa (S5 Appendix). Native and introduced *L*. *vulgare* plants were only partly separated (S5 Appendix). The PCA based on population means revealed a clearer separation of *L*. *vulgare* and *L*. *ircutianum* populations while the phenotypic spaces of native and introduced *L*. *vulgare* populations were still partially overlapping (Fig 2). *Leucanthemum vulgare* and *L*. *ircutianum* populations were mostly separated along the second axis while Eurasian and North American *L*. *vulgare* populations were separated along the first and second axis. The first axis of the PCA based on population means was most strongly positively correlated with the relative length of the teeth or lobes at the base of the stem leaf and negatively correlated with the length to width ratio of the stem leaf, the number of flower heads per plant, the biomass, the number of shoots and the perimeter to area ratio of the mid-stem leaves and the second axis was most strongly positively correlated with the length of the longest shoot at the end of the flowering stage, the blade length of the longest seedling leaf, the days to flowering and the diameter of the flower heads and negatively correlated with the perimeter to area ratio of the rosette leaf and the perimeter to area ratio of the stem leaf (S6 Appendix).

### Comparison of *Leucanthemum vulgare* and *L*. *ircutianum* from the native range

**Fig 3:**
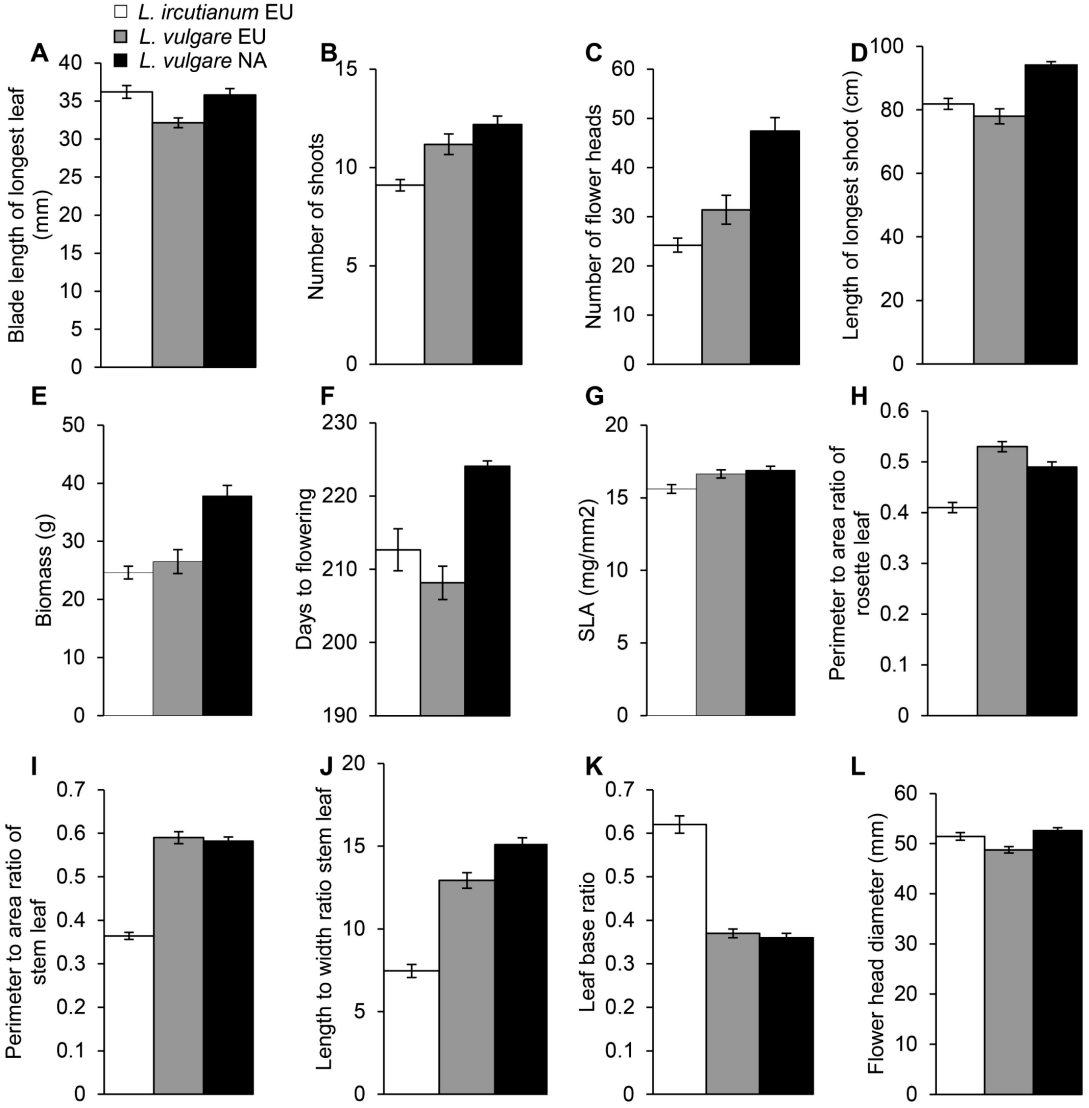
Mean and standard errors for (A) blade length of longest leaf at the seedling stage, (B) number of shoots per plant, (C) number of flower heads per plant, (D) length of longest shoot, (E) biomass at the end of the flowering stage, (F) days to flowering, (G) specific leaf area (SLA) of a rosette leaf, (H) perimeter to area ratio of a rosette leaf, (I) perimeter to area ratio of a stem leaf, (J) length to width ratio of a stem leaf (K) width of undivided middle part at the base devided by the total width at the base of a stem leaf and (L) flower head diameter for 21 native (Eurasia, EU) *Leucanthemum ircutianum* populations and 20 native and 21 invasive (North America, NA) *L*. *vulgare* populations grown under standardized conditions.

With regard to performance traits, germination rate was slightly lower for *L*. *vulgare* seeds than for those of *L*. *ircutianum* (*t* = 2.2, *P* = 0.04, S7 Appendix). The three-month old *L*. *vulgare* seedlings had shorter leaf blades (*t* = 3.9, df = 39, *P* < 0.001, Fig 3A), but the seedlings of the two species did not differ in their number of leaves (*z* = 0.1, *P* = 0.9, S7 Appendix). *Leucanthemum vulgare* produced on average 20% more shoots and flower heads than *L*. *ircutianum* (*z* = 3.6, *P* = 0.004 and *z* = 2.2, *P* = 0.03, Fig 3B and 3C) but, maximum height (*t* = 1.4, df = 39, *P* = 0.2, Fig 3D) and biomass at the end of the flowering stage (*t* = 0.7, df = 39, *P* = 0.5, Fig 3E) were similar for both species.

With regard to functional traits, days to germination (*z* = 0.4, *P* = 0.7, S7 Appendix) and days to flowering (*z* = 1.2, *P* = 0.2, Fig 3F) did not differ between native *L*. *vulgare* and *L*. *ircutianum*. SLA was 6% higher for *L*. *vulgare* than for *L*. *ircutianum* (*t* = 2.9, df = 39, *P* = 0.006, Fig 3G), but LDMC was similar for both species (*t* = 0.6, df = 39, *P* = 0.5, S7 Appendix).

In contrast to the moderate differences in performance and functional traits, the two species clearly differed in all of the measured leaf morphological traits, except for the length to width ratio of the leaf blades of the three-month old seedlings (*t* = 0.0, df = 39, *P* = 1.0, S7 Appendix): The perimeter to area ratio of the rosette and stem leaves was higher for *L*. *vulgare* compared to *L*. *ircutianum* and the stem leaves of *L*. *vulgare* had a larger length to width ratio and longer teeth and lobes at their base than those of *L*. *ircutianum* (all *P* < 0.001, Fig 3H and 3K). In addition, the flower heads of *L*. *vulgare* were slightly smaller compared to those of *L*. *ircutianum* (*t* = 2.6, df = 39, *P* = 0.01, Fig 3L).

### Comparison of native and introduced *Leucanthemum vulgare* populations

**Fig 4:**
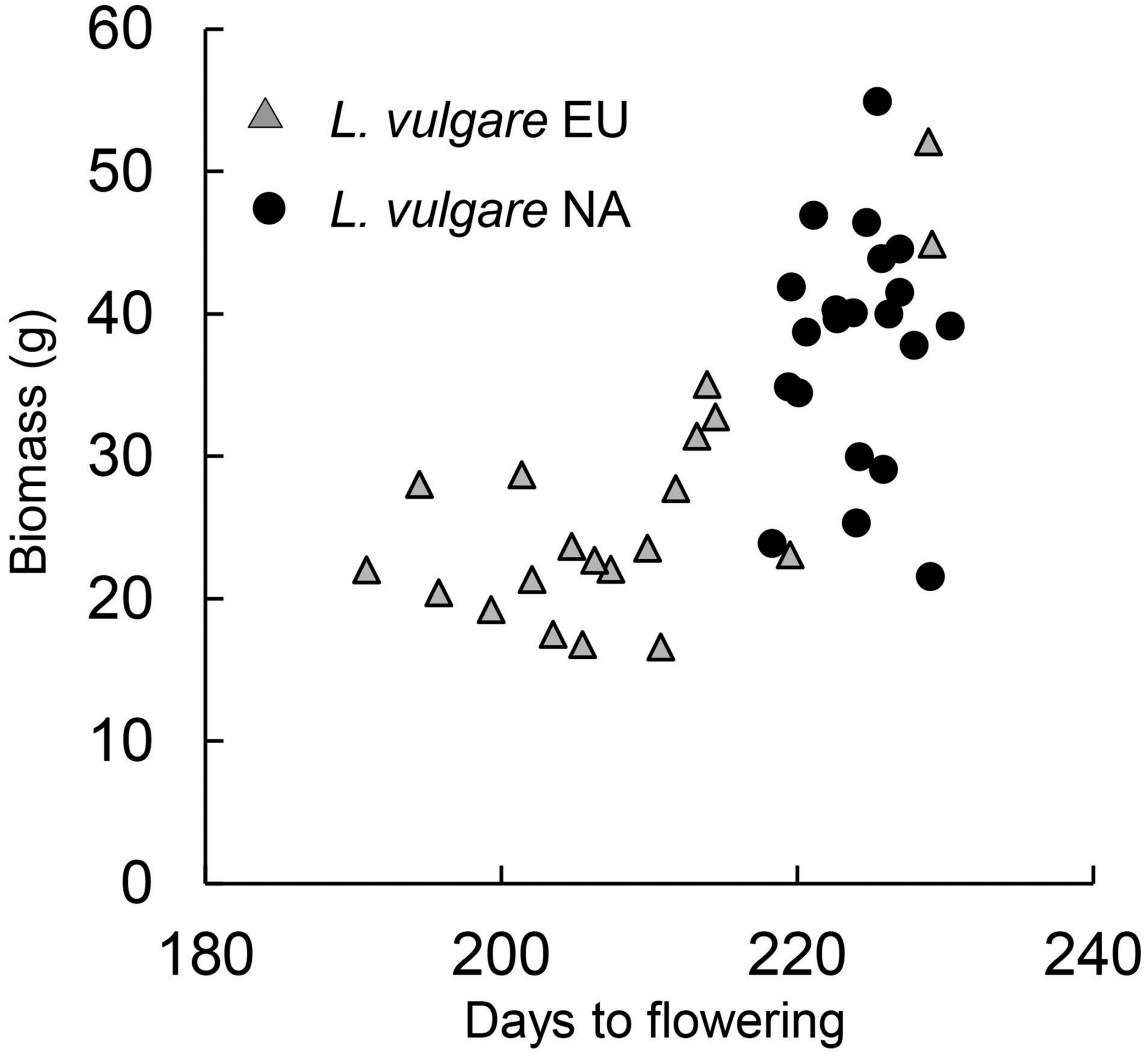
Relationship between days to flowering and biomass for native (Eurasia, EU, *t* = 3.7, df = 18, *P* = 0.002) and introduced (North America, NA, *t* = 0.3, df = 19, *P* = 0.8) *Leucanthemum vulgare* populations.

With regard to performance traits, germination rate and the number of leaves of three-month old seedlings were similar for native and introduced *L*. *vulgare* (*P* > 0.1, S7 Appendix), but the leaf blade of the longest leaf of the seedlings of introduced *L*. *vulgare* was on average longer than those of native *L*. *vulgare* (*t* = 3.5, df = 39, *P* = 0.001, Fig 3A) indicating that they had a higher biomass. At the end of the flowering stage, native and introduced *L*. *vulgare* had a similar number of shoots (*z* = 1.6, *P* = 0.1, Fig 3B), but total biomass of *L*. *vulgare* from the introduced range was 43% higher (Fig 3E), their longest shoot was 21% longer and they had 50% more flower heads (all *P* < 0.001, Fig 3C and 3D). With regard to functional traits, days to germination, LDMC and SLA did not differ between native and introduced *L*. *vulgare* (all *P >* 0.1, S7 Appendix, Fig 3G) but *L*. *vulgare* from the introduced range flowered on average 16 days later than those from the native range (*z* = 6.9, *P* < 0.001, Fig 3F). We found a positive correlation between biomass and days to flowering for native (*t* = 3.7, df = 18, *P* = 0.002) but not for introduced (*t* = 0.3, df = 19, *P* = 0.8) *L*. *vulgare* populations (Fig 4). The *L*. *vulgare* populations from the introduced range were collected from slightly more northern latitudes than those from the native range (*t* = 1.9, df = 39, *P* = 0.07). In the introduced range, biomass was negatively but not significantly correlated with latitude (*t* = 1.9, df = 19, *P* = 0.07), but there was no correlation between latitude and days to flowering (*t* = 1.3, df = 19, *P* = 0.2). Biomass and days to flowering of native *L*. *vulgare* populations were not correlated with latitude (both *P* > 0.1).

With regard to morphological traits, *L*. *vulgare* from the introduced range had larger flower heads (*t* = 5.4, df = 19, *P* < 0.001, Fig 3L) and their mid-stem leaves had a larger length to width ratio (*t* = 4.0, df = 19, *P* < 0.001, Fig 3J) than those from the native range, but they did not differ in any of the other leaf morphological traits (all *P >* 0.1).

## Discussion

Our common garden experiment revealed clear phenotypic differences between *L*. *vulgare* and *L*. *ircutianum* populations from the native range as well as between native and introduced *L*. *vulgare* populations. Native *L*. *vulgare* and *L*. *ircutianum* differed in morphological traits but not or only moderately in performance or functional traits that could explain the higher abundance of *L*. *vulgare* in its introduced range. In contrast, *Leucanthemum vulgare* populations from the introduced range flowered significantly later, were taller and had a higher biomass and more flower heads than those from the native range, suggesting that rapid evolution post-introduction may have contributed to the invasion success of *L*. *vulgare*. The leaf morphological traits, however, were similar for *L*.*vulgare* populations from the native and introduced range.

### Differences between *Leucanthemum vulgare* and *L*. *ircutianum* from the native range

We found that native *L*. *vulgare* had on average 20% more shoots and flower heads than *L*. *ircutianum*, but this did not result in a higher biomass, potentially due to their thinner stems, more dissected leaves and smaller flower heads. Similar to these results obtained under standardized conditions, a higher number of shoots and flower heads of *L*. *vulgare* compared to *L*. *ircutianum* was also observed in field populations [27]. The higher number of flower heads of native *L*. *vulgare* compared to *L*. *ircutianum* plants might have contributed to its higher invasion success if the number of flower heads per plant is closely related to reproductive output. However, since we did not record the number of seeds per plant it remains unclear whether the two species differ in the total number of seeds or whether the slightly smaller flower heads of *L*. *vulgare* resulted in similar numbers of seeds per plant of both species. Although it has been reported that field populations of *L*. *vulgare* would flower 1–2 weeks earlier than *L*. *ircutianum* [31], we found no difference in days to flowering between the two species under common garden conditions. We found that *L*. *vulgare* had a significantly higher SLA but similar LDMC compared to *L*. *ircutianum*. High SLA values have been associated with fast growth rates [50] and invasive species have repeatedly been found to have up to 20 to 30% higher SLAs than closely related non-invasive introduced species [5–7, 9, 10]. However, the relative difference in SLA between *L*. *vulgare* and *L*. *ircutianum* was only 6% and it is therefore questionable that this small difference is biologically relevant.

In contrast to the moderate differences in performance and functional traits between *L*. *vulgare* and *L*. *ircutianum* we found significant morphological differences between populations of the two species. In agreement with other studies (e.g. [40, 43]), *L*. *vulgare* had on average more dissected leaves with a higher leaf perimeter to area ratio, longer teeth or lobes at the base of the mid-stem leaves and a larger leaf length to width ratio than *L*. *ircutianum*. In addition, *L*. *vulgare* had slightly smaller flower heads than *L*. *ircutianum*. However, even though *L*. *vulgare* and *L*. *ircutianum* differed in several morphological traits, the perimeter to area ratio of the stem leaves was the only trait that clearly separated the two species at the population level and all of the traits measured showed overlapping values between the two species at the level of individual plants. This is in agreement with other studies that stated that multiple characters need to be considered in order to clearly distinguish the two species [42, 43].

Theoretically, the differences in leaf morphology found between *L*. *vulgare* and *L*. *ircutianum* may have contributed to the unequal invasion success of the two species. Highly dissected and narrow leaves, as found for *L*. *vulgare*, tend to have more effective heat loss and higher evaporation and assimilation rates than broad, undivided leaves, and this may be beneficial in warm, sun-exposed environments [51, 52], but it is unlikely that these differences in leaf shape played an important role in the invasion success of *L*. *vulgare* since *L*. *vulgare* is also invasive in regions with high precipitation, such as western parts of Washington and Oregon and in regions with low temperatures, such as Alberta and British Columbia.

The observed phenotypic differences between the diploid *L*. *vulgare* and the tetraploid *L*. *ircutianum* may potentially be due to their differences in ploidy level. Polyploidy has often been associated with larger plants that produce larger leaves and fewer but larger flowers and seeds [53–56]. In addition, later germination, slower growth rates and later flowering as well as increased early growth rates have been found for polyploids compared to diploids [53–56]. Most of these phenotypic traits were similar for *L*. *vulgare* and *L*. *ircutianum* and only the larger flower heads of *L*. *ircutianum* are in line with these general effects of polyploidy. In agreement with our results, a similar study which compared diploid and allotetraploid cytotypes of *Centaurea stoebe* under standardized conditionsfound that the tetraploid cytotype had less dissected leaves and a lower SLA compared to the diploid cytotype [24]. However, because of the allopolyploid origin of *L*. *ircutianum* [33], hybridization or post-polyploidization evolution may have also contributed to the phenotypic differences between *L*. *vulgare* and *L*. *ircutianum*, potentially masking proposed phenotypic consequences of polyploidization.

Our common garden study provides little evidence that innate phenotypic traits pre-disposed *L*. *vulgare* to become more abundant in North America than *L*. *ircutianum*. However, the results of common garden studies can vary with the location and environmental conditions of the study [11, 57]. The environmental conditions experienced in our study are likely to be different than those in the introduced range and phenotypic traits may therefore be expressed differently than in the introduced range. To further elucidate whether *L*. *vulgare* possesses traits that particularly contribute to its invasion success common garden studies at multiple sites in the native and introduced range or common garden experiments that are more closely mimicking the environmental conditions encountered in the invaded habitats (e.g. regarding soil type and interacting species) need to be conducted.

### Differences between native and introduced *Leucanthemum vulgare*

We found that *L*. *vulgare* plants from the introduced range flowered significantly later, were taller and had a higher biomass and more flower heads than those from the native range. These results are in agreement with those found in a field study comparing *L*. *vulgare* populations in the US and in Europe [27]. Because of a potential trade-off between size and age of reproduction [58, 59], the higher biomass of introduced *L*. *vulgare* may be a consequence of their later flowering. In line with this, we found a positive correlation between days to flowering and biomass for native *L*. *vulgare* populations, but not for introduced ones. A few other common garden studies also found a shift in days to flowering between native and introduced populations. For example, similar to our study, introduced North American populations of *Centaurea diffusa* Lam. flowered later and plants were larger than native European ones [16] and introduced populations of *Ambrosia artemisiifolia* L flowered later compared to native conspecifics [60]. In contrast, earlier flowering of introduced compared to native populations was found for *Centaurea stoebe* L., *C*. *solstitialis* L. and *Silene latifolia* Poir. [21, 61–64].

Differences in the days to flowering may evolve as an adaptation to differences in land use practices. Several plant species have been found to adapt their flowering phenology according to the time of mowing or grazing [65–67]. For instance, van Tienderen and van der Toorn [65] conducted a reciprocal-transplant experiment with *Plantago lanceolata* L. and found that plants from an early-mown hayfield flowered earlier than those from a late-mown hayfield and that plants from a pasture population flowered last across all transplantation sites. Similarly, Reisch and Poschlod [66] found that under common garden conditions *Scabiosa columbaria* L. populations from mown sites flowered on average four weeks earlier than those from grazed sites. *Leucanthemum vulgare* is generally avoided by cattle [28], but the mowing regime may highly affect their flowering time. Based on observations made during field surveys to study the herbivore communities associated with *L*. *vulgare* in Europe and North America [27], the mowing regime of sites with *L*. *vulgare* appears to differ between the two ranges. In Europe, *L*. *vulgare* and *L*. *ircutianum* mainly occur on meadows and pastures, most of which are generally mown for a first time in June and are not commonly found on sites that are not regulary mown. In North America, however, *L*. *vulgare* is often found on sites such as waste areas, landfills and forests, which are not mown, or on meadows, pastures and roadsides (S2 Appendix) that are mown later in the season. Consequently, in Europe, there is likely a strong selection pressure for early flowering while in North America there may be selection for late flowering plants as they have the advantage to grow larger and produce more seeds. Interestingly, two *L*. *vulgare* populations from the native range (DE8 from Germany and GE1 from Georgia) flowered later and had a higher biomass compared to all other native populations and also compared to the average introduced population. The seeds from these two populations had been collected from ruderal habitats that had not been mown in the past while most of the other seeds from the native range had been collected from sites that were mown at least once per year.

Differences in the days to flowering between the native and introduced range could potentially also result from an adaptation to differences in climate. For several native and invasive plant species grown under standardized conditions, populations collected from more northern latitudes were found to flower earlier and at a smaller size than those collected from southern locations (e.g. [11, 68–74]). Hence, phenotypic differences between ranges can be confounded with latitudinal clines [75]. *Leucanthemum vulgare* populations from the introduced range were collected from slightly more northern latitudes than those from the native range and no correlation between days to flowering or biomass and latitude was found, neither for *L*. *vulgare* from the native nor for those from the introduced range. Therefore, the later flowering of North American *L*. *vulgare* is unlikely caused by climatic differences between the two ranges. In addition, differences in the days to flowering between the native and introduced range might hypothetically also be the result of different selection pressures imposed by pre-dispersal seed predators or by pollinators between the two ranges. Both groups of organisms have been found to impose selection pressure on the time of flowering of plants and generally, pollinators tend to promote early flowering whereas seed predators tend to promote late flowering [76].

In contrast to traits affecting performance, most of the morphological traits did not differ between *L*. *vulgare* populations from the native and introduced range. The only two morphological traits that significantly differed between native and introduced populations of *L*. *vulgare* were a larger length to width ratio of the mid-stem leaves and a larger size of the flower heads. The larger length to width ratio of mid-stem leaves of introduced compared to native *L*. *vulgare* was due to an increase by 17% in leaf length at a similar leaf width and is therefore, together with the larger size of the flower heads, likely a consequence of their overall larger size.

Besides selection in the introduced range, genetic drift due to founder effects could also be responsible for the observed differences between native and introduced populations of *L*. *vulgare*. However, *L*. *vulgare* has been introduced multiple times to North America as ornamental and as seed contaminant [28] and it is very unlikely that the introduced plants exclusively consisted of late-flowering genotypes. Therefore, we suggest that natural selection in the introduced range more likely explains the observed pattern than founder effects. Since late-flowering *L*. *vulgare* populations were also found in the native range, they were potentially also among the plants introduced to North America, but late flowering genotypes may also have evolved in the introduced range independently from the native range. To discriminate between these hypotheses comprehensive molecular studies would be necessary.

Because we grew plants from field-collected seeds, environmental maternal effects could also have contributed to the observed differences between native and introduced *L*. *vulgare* populations [77]. Hence, their influence on the performance of *L*. *vulgare* populations collected in different biogeographic regions should be considered in future studies. However, maternal effects tend to mainly affect early development and their effects often decrease later in the life cycle [77–81]. Hence, while maternal effects may play a role in explaining the differences in leaf size of three-month old seedlings between native and introduced *L*. *vulgare*, they are unlikely responsible for the large differences in days to flowering and biomass at the end of flowering.

## Conclusions

Our study did not provide clear evidence that *L*. *vulgare* was better pre-disposed to become invasive than *L*. *ircutianum* and we suggest that future studies should focus on identifying alternative explanations for the higher invasion success of *L*. *vulgare* compared to *L*. *ircutianum*. However, our results support our hypothesis that rapid evolution in the introduced range has likely contributed to the invasion success of *L*. *vulgare* in North America. One possible explanation for the increased performance of introduced compared to native *L*. *vulgare* populations is a release from a trade-off between early flowering and size due to a less intensive mowing regime of *L*. *vulgare* habitats in the introduced range. Our study contributes to the growing evidence showing that rapid evolution in the introduced range is common and that adaptation to altered environmental conditions may contribute to the invasion success of introduced plants [13, 14].

## Supporting information

S1 Appendix Results of flow cytometric analyses conducted with *Leucanthemum* seeds purchased from twelve US and one Canadian seed company. (PDF)

S2 Appendix Locations of *Leucanthemum vulgare* and *L*. *ircutianum* populations sampled in Eurasia and North America. (PDF)

S3 Appendix List of traits recorded for *Leucanthemum vulgare* and *L*. *ircutianum* grown in a common garden. (PDF)

S4 Appendix Measurements taken on scanned mid-stem leaves of *Leucanthemum vulgare* (left) and *L*. *ircutianum* (right). (PDF)

S5 Appendix Principal Component Analysis (PCA) plots based on 17 traits measured on 536 plants of 62 *Leucanthemum vulgare* and *L*. *ircutianum* populations from the native (Eurasia, EU) and introduced (North America, NA) range grown in a common garden. (PDF)

S6 Appendix Eigenvectors showing correlations of traits with the first two principal components for the Principal Component Analyses (PCAs) on individual plants (see S4 Appendix) and on population means (see Fig 2). (PDF)

S7 Appendix Mean (± SE) trait values for 20 *Leucanthemum vulgare* and 21 *L*. *ircutianum* populations from the native range, Eurasia (EU) and 21 *L*. *vulgare* populations from the introduced range, North America (NA) grown in a common garden. (PDF)

S1 Data (TXT)
