# Supplementary material for: Biological invasion of oxeye daisy (Leucanthemum vulgare) in North America: Pre-adaptation, post-introduction evolution, or both?
Source: PLoS One. 2018 Jan 4;13(1):e0190705. doi: 10.1371/journal.pone.0190705 (PMC5754128; doi:10.1371/journal.pone.0190705)
Supplement: S1 Appendix — (PDF) [file pone.0190705.s001.pdf]

S1 Appendix. Results of flow cytometric analyses conducted with *Leucanthemum* seeds purchased from twelve US and one Canadian seed company. Two samples, each containing ten seeds were analysed from each seed source (for more information on the methods see materials and methods section in the main article).

Seed Company	Country	Species name on package	Ploidy level	Correct species name
Blooming flower seeds	USA	Oxeye daisy, *Chrysanthemum leucanthemum*	4x	*Leucanthemum ircutianum*
Buy Wholesale cheap	USA	Oxeye Daisy	4x	*Leucanthemum ircutianum*
Dollarseed	USA	Oxeye Daisy	4x	*Leucanthemum ircutianum*
Eden Brothers	USA	Oxeye daisy, *Chrysanthemum leucanthemum*	4x	*Leucanthemum ircutianum*
Everwilde Farms	USA	Oxeye daisy, *Chrysanthemum leucanthemum*	4x	*Leucanthemum ircutianum*
Horizon Herbs	USA	Oxeye daisy, *Chrysanthemum leucanthemum*	2x	*Leucanthemum vulgare*
Sand Mountain Herbs	USA	Oxeye daisy, *Chrysanthemum leucanthemum*	4x	*Leucanthemum ircutianum*
Seedterra	USA	Oxeye daisy, *Chrysanthemum leucanthemum*	4x	*Leucanthemum ircutianum*
Seedville	USA	Oxeye daisy, *Chrysanthemum leucanthemum*	4x	*Leucanthemum ircutianum*
Sheffield's Seed	USA	Oxeye Daisy, *Leucanthemum vulgare*	4x	*Leucanthemum ircutianum*
Todd's seed	USA	Oxeye daisy, *Chrysanthemum leucanthemum*	4x	*Leucanthemum ircutianum*
Top Tropicals	USA	*Leucanthemum vulgare*	4x	*Leucanthemum ircutianum*
Richters Herbs	Canada	Oxeye daisy, *Chrysanthemum leucanthemum*	4x	*Leucanthemum ircutianum*
