# Supplementary material for: Biological invasion of oxeye daisy (Leucanthemum vulgare) in North America: Pre-adaptation, post-introduction evolution, or both?
Source: PLoS One. 2018 Jan 4;13(1):e0190705. doi: 10.1371/journal.pone.0190705 (PMC5754128; doi:10.1371/journal.pone.0190705)
Supplement: S2 Appendix — (PDF) [file pone.0190705.s002.pdf]

S2 Appendix. Locations of *Leucanthemum vulgare* and *L. ircutianum* populations sampled in Eurasia and North America.

Population code	Country / state	Species	Latitude (°N)	Longitude (°W)	Elevation (m)	Habitat
Native range (Eurasia)						
BE1	Belgium	*L. vulgare*	50.1091	5.2194	212	roadside
CZ04	Czech Republic	*L. vulgare*	49.2381	15.6708	474	forest/roadside
CZ22	Czech Republic	*L. vulgare*	50.3513	15.7563	265	meadow
CZ28	Austria	*L. vulgare*	48.9174	15.3221	468	meadow
CZ30	Austria	*L. vulgare*	48.7266	14.9107	511	meadow
DE8	Germany	*L. vulgare*	48.2254	8.7720	643	ruderal
DE9	Germany	*L. vulgare*	49.0406	11.8897	460	field edge
ES14	Spain	*L. vulgare*	42.3636	1.2987	1326	roadside
FR17	France	*L. vulgare*	43.9667	3.1185	782	pasture
FR32	France	*L. vulgare*	44.0671	3.2002	483	pasture
FR33	France	*L. vulgare*	44.1473	3.2680	821	meadow
FR34	France	*L. vulgare*	44.1301	3.4001	974	meadow
FR38	France	*L. vulgare*	44.2891	3.3329	848	roadside
FR44	France	*L. vulgare*	43.8404	3.1400	632	pasture
FR46	France	*L. vulgare*	43.9401	3.0089	542	meadow
GE1	Georgia	*L. vulgare*	41.5729	44.3779	1477	pipeline corridor
GE4	Georgia	*L. vulgare*	41.6473	42.4787	1580	n/a
HU	Hungary	*L. vulgare*	47.4158	19.8206	105	meadow
PL3VUL	Poland	*L. vulgare*	49.5829	19.9350	557	roadside
RO5	Romania	*L. vulgare*	47.4748	26.2703	375	pasture
BE2	Belgium	*L. ircutianum*	50.1534	5.4179	335	roadside
CH5	Switzerland	*L. ircutianum*	46.3495	8.7866	910	meadow
CH6	Switzerland	*L. ircutianum*	47.3238	7.3498	587	meadow
CZ2	Czech Republic	*L. ircutianum*	50.7859	15.1564	664	meadow
CZ9	Czech Republic	*L. ircutianum*	49.2051	17.8553	383	meadow
CZ21	Czech Republic	*L. ircutianum*	50.3825	15.7380	281	meadow
CZ29	Austria	*L. ircutianum*	48.7824	15.0382	522	meadow
DE6	Germany	*L. ircutianum*	48.1688	8.3945	903	meadow
FR20	France	*L. ircutianum*	43.4395	2.3973	600	meadow
FR26	France	*L. ircutianum*	47.3241	-2.0137	6	meadow
FR36	France	*L. ircutianum*	44.1108	3.4036	750	meadow
FR39	France	*L. ircutianum*	43.9678	3.1144	808	meadow
FR41	France	*L. ircutianum*	43.7559	2.9761	744	pasture
FR43	France	*L. ircutianum*	43.4340	2.6053	801	pasture
GE2	Georgia	*L. ircutianum*	41.7388	43.5004	1654	roadside/meadow
PL3IRC	Poland	*L. ircutianum*	49.5829	19.9340	557	roadside
RO6	Romania	*L. ircutianum*	47.0949	26.3254	259	meadow
RO8	Romania	*L. ircutianum*	45.3321	22.7253	1096	roadside/meadow
RUS2	Russia	*L. ircutianum*	60.0650	30.4365	19	roadside
RUS3	Russia	*L. ircutianum*	50.5415	36.0395	200	meadow
SK2	Slovakia	*L. ircutianum*	48.8795	19.2333	963	roadside

Population code	Country / state	Species	Latitude (N)	Longitude (W)	Elevation (m)	Habitat
Introduced range (Canada, USA)						
NA1	British Columbia	*L. vulgare*	54.9485	-127.2445	480	roadside
NA3	Alberta	*L. vulgare*	51.1781	-115.7164	1472	roadside
NA4	British Columbia	*L. vulgare*	51.5541	-121.2682	1186	utility line
NA5	British Columbia	*L. vulgare*	49.9632	-116.9750	1167	roadside
NA6	British Columbia	*L. vulgare*	53.8645	-123.2503	796	roadside
NA7	British Columbia	*L. vulgare*	48.5690	-123.6368	235	roadside
NA8	British Columbia	*L. vulgare*	48.5138	-123.5717	378	roadside
NA10	Montana	*L. vulgare*	46.9564	-112.7105	1375	pasture
NA11	Minnesota	*L. vulgare*	46.1726	-92.8709	338	pasture
NA12	Wyoming	*L. vulgare*	43.4980	-110.8520	1890	roadside/meadow
NA13	Minnesota	*L. vulgare*	45.2010	-92.8837	298	roadside
NA14	Minnesota	*L. vulgare*	47.2060	-91.3685	217	roadside
NA15	Colorado	*L. vulgare*	39.1667	-105.1655	2246	riperian
NA16	Washington	*L. vulgare*	47.3032	-121.2878	750	forest opening
NA17	Washington	*L. vulgare*	47.0585	-123.1941	115	forest opening/roadside
NA18	Washington	*L. vulgare*	46.8840	-123.3069	38	meadow
NA19	Oregon	*L. vulgare*	45.1753	-121.6784	1115	ruderal
NA20	Montana	*L. vulgare*	46.6995	-116.5402	835	Conservation Reserve Program
NA21	Montana	*L. vulgare*	47.3221	-115.0760	887	meadow
NA22	British Columbia	*L. vulgare*	49.5016	-114.2797	1283	meadow
NA23	Alberta	*L. vulgare*	54.5906	-113.2501	624	field edge
