# Supplementary material for: Biological invasion of oxeye daisy (Leucanthemum vulgare) in North America: Pre-adaptation, post-introduction evolution, or both?
Source: PLoS One. 2018 Jan 4;13(1):e0190705. doi: 10.1371/journal.pone.0190705 (PMC5754128; doi:10.1371/journal.pone.0190705)
Supplement: S3 Appendix — (PDF) [file pone.0190705.s003.pdf]

S3 Appendix. List of traits recorded for *Leucanthemum vulgare* and *L. ircutianum* grown in a common garden.

Code	Description	Reason why this trait was included in the study
**Performance traits**		
germrate	Germination rate	Correlated with population performance (establishment success and spread)
bladeL	Blade length of longest leaf of three-month old seedlings (mm)	Correlated with seedling biomass, indication for seedling growth, competitive ability
leaves	Number of leaves longer than 1 cm at the seedling stage	Indicator of seedling growth, competitive ability
shoots	Number of shoots at the end of the flowering stage	Correlated with plant size, vegetative performance, competitive ability
FH	Number of flower heads at the end of the flowering stage	Correlated with reproductive output/fitness
shootlength	Length of longest shoot at the end of the flowering stage (cm)	Correlated with plant size, vegetative performance, competitive ability
biomass	Dry weight of above ground biomass at the end of the flowering stage (g)	Correlated with plant size, vegetative performance, competitive ability
**Functional traits**		
germtime	Number of days from sowing to germination	Rapid germination may increase establishment through size advantage
sow_flow	Number of days from sowing to first flower completely open	Potential differences between species (*L. vulgare* is reported to flower earlier than *L. ircutianum*) and ranges (in the field *L. vulgare* is observed to flower later in North America than in Europe)
LDMC	Leaf dry matter content: ratio of dry to fresh weight of a randomly chosen rosette leaf	Indicator of resource-use strategy, correlated with resource conservation, negatively correlated with relative growth rate
SLA	Specific leaf area: ratio of area to dry weight of a randomly chosen rosette leaf (mm^2/mg)	Indicator of resource-use strategy, correlated with relative growth rate
**Morphological traits**		
bladeL_bladeW	Length to width ratio of the blade of the longest leaf of three-month old seedlings	Potentially important for species determination at seedling stage
ros_peri_area	Perimeter to area ratio of randomly chosen rosette leaf	Indicator of leaf dissection, potentially important for species determination at rosette stage
st_peri_area	Perimeter to area ratio of randomly chosen mid-stem leaf	Indicator of leaf dissection, potentially important for species determination at flowering stage
st_length_width	Length to width ratio of randomly chosen mid-stem leaf (leaf width was measured at the leaf length midpoint, not including lobes and teeth)	Potentially important for species determination at flowering stage
st_mid_base	Width of undivided middle part at the base devided by the total width at the base of a randomly chosen stem leaf	Potentially important for species determination at flowering stage
flowdia	Flower head diameter (mm, average taken from three flower heads per plant)	Potentially important for species determination at flowering stage
