# Supplementary material for: Biological invasion of oxeye daisy (Leucanthemum vulgare) in North America: Pre-adaptation, post-introduction evolution, or both?
Source: PLoS One. 2018 Jan 4;13(1):e0190705. doi: 10.1371/journal.pone.0190705 (PMC5754128; doi:10.1371/journal.pone.0190705)
Supplement: S4 Appendix — (PDF) [file pone.0190705.s004.pdf]

**S4 Appendix.** Measurements taken on scanned mid-stem leaves of *Leucanthemum vulgare* (left) and *L. ircutianum* (right).

A: leaf length

B: leaf width, measured at the leaf length midpoint, not including any lobes or teeth

C: width of undivided middle part at the leaf base

D: total width at the leaf base

**st_length_width:** A/D

**st_mid_base:** C/D

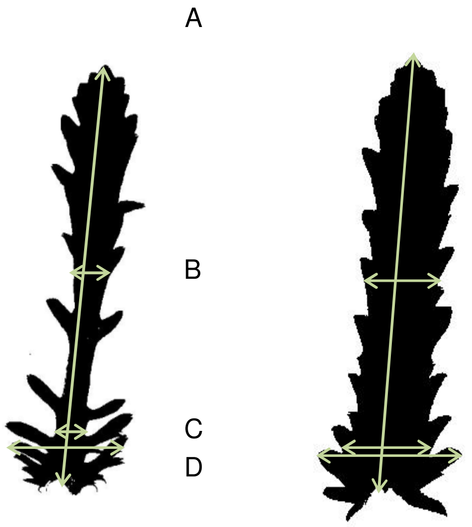


*L. vulgare* *L. ircutianum*

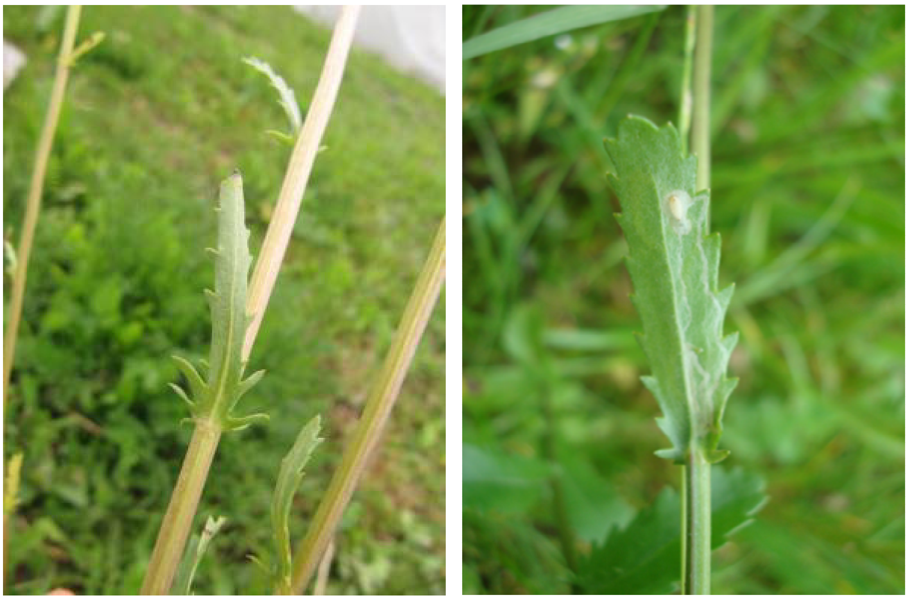

Pictures of a typical mid-stem leaf of *L. vulgare* (left) and *L. ircutianum* (right).
