# Supplementary material for: Biological invasion of oxeye daisy (Leucanthemum vulgare) in North America: Pre-adaptation, post-introduction evolution, or both?
Source: PLoS One. 2018 Jan 4;13(1):e0190705. doi: 10.1371/journal.pone.0190705 (PMC5754128; doi:10.1371/journal.pone.0190705)

S5 Appendix. Principal Component Analysis (PCA) plots based on 17 traits measured on 556 plants of 62 *Leucanthemum vulgare* and *L. ircutianum* populations from the native (Eurasia, EU) and introduced (North America, NA) range grown in a common garden. To show the phenotypic space occupied by the species from the native and introduced range confidence ellipses defined by the gravity center (centroid) of the cloud and 1.5 times the standard deviation were constructed. The first axis explains 20.1% of total variation in the dataset; the second axis explains 13.9%.

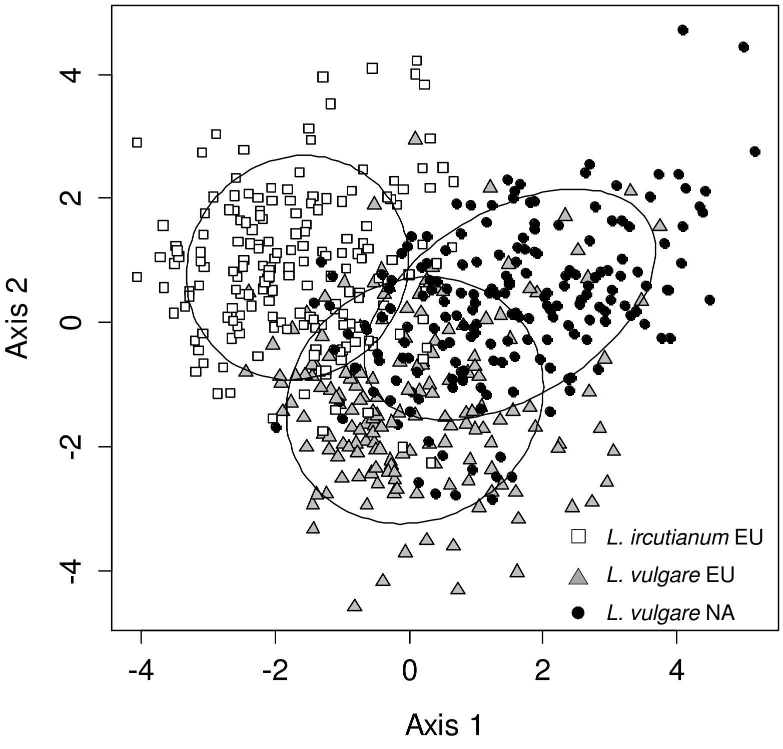

Supplement: S5 Appendix — (PDF) [file pone.0190705.s005.pdf]
