# Supplementary material for: Biological invasion of oxeye daisy (Leucanthemum vulgare) in North America: Pre-adaptation, post-introduction evolution, or both?
Source: PLoS One. 2018 Jan 4;13(1):e0190705. doi: 10.1371/journal.pone.0190705 (PMC5754128; doi:10.1371/journal.pone.0190705)
Supplement: S6 Appendix — (PDF) [file pone.0190705.s006.pdf]

S6 Appendix. Eigenvectors showing correlations of traits with the first two principal components for the Principal Component Analyses (PCAs) on individual plants (see Appendix S4) and on population means (see Fig. 2). Values for traits best correlated with the first or second PCA axis (> 0.30) are in bold. For the abbreviations of the different traits see Appendix S2.

	PCA based on plants		PCA based on populations	
Trait	Axis 1	Axis 2	Axis 1	Axis 2
biomass	**0.393**	**0.311**	**-0.346**	0.264
bladeL	0.041	**0.347**	0.035	**0.390**
bladeL_bladeW	-0.004	0.120	0.063	0.048
FH	**0.426**	0.203	**-0.373**	0.170
flowdia	0.003	0.247	-0.000	**0.325**
germrate	-0.119	0.051	0.212	0.028
germtime	0.072	-0.107	-0.154	-0.025
LDMC	-0.129	0.059	0.097	0.180
leaves	0.071	0.047	0.045	0.015
ros_peri_area	0.202	**-0.361**	-0.218	**-0.360**
shootlength	0.265	**0.367**	-0.270	**0.395**
shoots	**0.333**	0.039	**-0.317**	0.005
SLA	0.192	-0.084	-0.195	-0.094
sow_flow	0.198	0.276	-0.219	**0.375**
st_length_width	**0.355**	-0.140	**-0.387**	-0.055
st_mid_base	**-0.335**	**0.335**	**0.329**	0.269
st_peri_area	0.293	**-0.405**	**-0.302**	**-0.311**
