# Supplementary material for: Biological invasion of oxeye daisy (Leucanthemum vulgare) in North America: Pre-adaptation, post-introduction evolution, or both?
Source: PLoS One. 2018 Jan 4;13(1):e0190705. doi: 10.1371/journal.pone.0190705 (PMC5754128; doi:10.1371/journal.pone.0190705)
Supplement: S7 Appendix — (PDF) [file pone.0190705.s007.pdf]

S7 Appendix. Mean (± SE) trait values for 20 *Leucanthemum vulgare* and 21 *L. ircutianum* populations from the native range, Eurasia (EU) and 21 *L. vulgare* populations from the introduced range, North America (NA) grown in a common garden. For the abbreviations of the different traits see Appendix S2.

Trait	*L. ircutianum* (EU)	vs.[a]	*L. vulgare* (EU)	vs.[a]	*L. vulgare* (NA)
**Performance traits**					
germrate	0.88 ± 0.02	0.04	0.79 ± 0.04	n.s.	0.80 ± 0.02
bladeL	36.20 ± 0.84	<0.001	32.14 ± 0.63	0.001	35.80 ± 0.83
leaves	11.40 ± 0.35	n.s.	11.25 ± 0.40	n.s.	11.82 ± 0.33
shoots	9.10 ± 0.29	0.004	11.18 ± 0.53	n.s.	12.18 ± 0.43
FH	24.20 ± 1.43	0.03	31.40 ± 2.91	<0.001	47.38 ± 2.75
shootlength	81.86 ± 1.73	n.s.	77.98 ± 2.36	<0.001	94.08 ± 1.09
biomass	24.62 ± 1.11	n.s.	26.50 ± 2.06	<0.001	37.83 ± 1.80
**Functional traits**					
germtime	9.20 ± 0.26	n.s.	9.54 ± 0.52	n.s.	9.70 ± 0.28
sow_flow	212.66 ± 2.88	n.s.	208.15 ± 2.27	<0.001	224.06 ± 0.73
LDMC	0.15 ± 0.00	n.s.	0.15 ± 0.00	n.s.	0.15 ± 0.00
SLA	15.61 ± 0.30	0.006	16.64 ± 0.28	n.s.	16.91 ± 0.25
**Morphological traits**					
bladeL_bladeW	1.34 ± 0.08	n.s.	1.34 ± 0.02	n.s.	1.32 ± 0.02
ros_peri_area	0.41 ± 0.01	<0.001	0.53 ± 0.01	n.s.	0.49 ± 0.01
st_peri_area	0.36 ± 0.01	<0.001	0.59 ± 0.01	n.s.	0.58 ± 0.01
st_length_width	7.46 ± 0.39	<0.001	12.93 ± 0.47	<0.001	15.10 ± 0.41
st_mid_base	0.62 ± 0.02	<0.001	0.37 ± 0.01	n.s.	0.36 ± 0.01
flowdia	51.43 ± 0.76	0.01	48.77 ± 0.64	<0.001	52.59 ± 0.58

[a] *P*-values from linear mixed models and generalized linear mixed models comparing Eurasian (EU) *L. vulgare* with Eurasian *L. ircutianum* populations and Eurasian with North American (NA) *L. vulgare* populations. n.s.: $P > 0.05$.
